# Larvicidal and Repellent Activity of *Mentha arvensis* L. Essential Oil against *Aedes aegypti*

**DOI:** 10.3390/insects11030198

**Published:** 2020-03-22

**Authors:** Ho Dung Manh, Ong Thi Tuyet

**Affiliations:** 1 Department of Pharmaceutical Chemistry, Faculty of Pharmacy, Lac Hong University, Dong Nai 810000, Vietnam; 2 Department of Traditional Medicine, Faculty of Pharmacy, Lac Hong University, Dong Nai 810000, Vietnam; tuyetong2610@gmail.com

**Keywords:** *Mentha arvensis*, essential oil, *Aedes aegypti*, Larvicidal activity, mosquito repellent

## Abstract

Dengue is one of the most dangerous vector-borne diseases transmitted by *Aedes* mosquitoes. The use of mosquito repellents to protect human hosts and insecticides to reduce the mosquito population is a crucial strategy to prevent the disease. Here, we reported larvicidal and repellent activities of *Mentha arvensis* L. essential oil against *Aedes aegypti*, the main vector of the disease. The essential oil was extracted by hydro-distillation from the aromatic plant grown in Vietnam. The yield was 0.67% based on the weight of fresh leaves. The essential oil was analyzed by gas chromatography-mass spectrometry (GC-MS). The main components were menthol (66.04%), menthyl acetate (22.19%), menthone (2.51%), and limonene (2.04%). Toxicity test on *Aedes aegypti* larvae showed that the median lethal concentrations, LC_50_ and LC_90_ were 78.1 ppm (part per million) and 125.7 ppm, respectively. Besides, the essential oil showed excellent repellency on *Aedes aegypti* mosquitoes. At 25%, 50%, and 100% concentration, the respective complete protection times (CPTs) were 45 min, 90 min, and 165 min. When adding 5% vanillin to the essential oil (25%), the complete protection time of the essential oil increased up to 120 min. In conclusion, the EO from *Mentha arvensis* L. has been shown to be a promising natural larvicide and repellent against *Aedes aegypti* mosquitoes.

## 1. Introduction

Dengue is one of the most important vector-borne diseases and is transmitted by *Aedes* mosquitoes. There are millions of infections that occur every year in the world [1,2]. Global warming and human population growth have led to an increase in mosquito population and number of infections [3]. Disease control in practice usually includes using insecticide to reduce the mosquito population [4] and using chemical repellents to protect the human host from mosquito bites [5].

Although synthetic insecticides such as organophosphate, pyrethroid, etc. have reduced the *Aedes* mosquito population successfully, the continuous increase in the use of the synthetic insecticides has led to mosquito resistance [6], and more importantly, potential toxicity in the environment and adverse effects on human health [7,8]. Besides, repellents are often used to protect against mosquito bites by applying on human skin. One of the most effective chemical repellents is DEET (N,N-diethyl-3-methylbenzamide), which is widely used in commercial products [9]. However, DEET has some potential risks for human health, especially a high level of DEET was reported to have adverse effects on children [10].

Many recent studies have been focused on plant-based products that present low toxicity and reduce the accumulation of toxic chemicals in the environment [11,12]. Among these natural products, essential oils have been shown to be potential alternatives to synthetic chemicals because they are effective, eco-friendly and available to many parts in the world affected by mosquito vector-borne disease [13]. For example, common ingredients used in mosquito repellents are citronella oil, lemongrass oil, and para-menthane 3,8-diol (PMD) [14] found in waste distillate after extraction of the eucalyptus citriodora oil. 

In this study, we aim to extract the essential oil from the *Mentha arvensis* L. aromatic plant, grown in southern Vietnam, and to evaluate the larvicidal and repellent activities of the essential oil against *Aedes aegypti* mosquitoes.

## 2. Materials and Methods

### 2.1. Essential oil Extraction

*Mentha arvensis* L. plant was collected in Ho Chi Minh city, in July 2018. The plant was identified by a botanist, and a voucher specimen (1903) was deposited at the Faculty of Pharmacy, Lac Hong University, Vietnam. Two hundred grams of the fresh plant leaves were hydro-distilled by using a Clevenger apparatus. The oil layer was separated, and subsequently dried over anhydrous Na_2_SO_4_. Finally, the essential oil was stored in a sealed glass vial and further analyzed using GC-MS.

### 2.2. GC/MS

The essential oil was characterized and quantified by GC–MS analysis on an Agilent 6890N gas chromatograph instrument equipped with an Agilent 5973 mass spectrometer and an HP-5MS capillary column (length 30 m × 0.25 mm ID, film thickness 0.25 mm; Agilent-Technologies, Palo Alto, CA, USA). The carrier gas was helium at a constant flow of 1.0 mL/min. The oven temperature programs were as follows: from 50 °C (held for 2 min) to 80 °C (2 °C/min), from 80 °C to 150 °C (5 °C/min), from 150 °C to 200 °C (10 °C/min), from 200 °C to 300 °C (20 °C/ min) and held there for 5 min. The temperature of injector was 250 °C. The samples were diluted in hexane (1:40 v/v), then 1 μL of the diluted samples were injected in splitless mode. Component identification was done based on MS library search (NIST and Wiley). The percentage composition was calculated by integrating the peak areas of the chromatograms. 

### 2.3. Mosquito Rearing

The colony of *Aedes aegypti* mosquitoes was reared in the insectary at the Faculty of Pharmacy, Lac Hong University using the standard procedures described by Manh et al. [15,16]. The insectary was kept at 27 ± 3 °C, 70%–80% relative humidity with a photoperiod of 12 h light and 12 h dark. Larvae were placed in plastic trays and provided with cat food (Wiskcat), whereas adult mosquitoes were kept in breeding cages (30 cm × 30 cm × 30 cm) and maintained on a 10% sucrose solution. The female mosquitoes were fed with blood of live mice for mosquito reproduction. These studies were conducted following the Guide for the Care and Use of Laboratory Animals of Faculty of Pharmacy, Lac Hong University.

### 2.4. Larvicidal Assay

The larvicidal activity was tested based on the recommendations of WHO [17]. The essential oil was diluted in 1ml ethanol and then diluted in tap water to obtain 100 mL of serial solutions of different concentrations: 45, 60, 75, 90, 115, 120 part per million (ppm). Control solutions were made with 1 mL of alcohol in 99 mL of tap water. Batchs of 25 third or fourth instar larvae were transferred by pasteur pipette to cups containing a 100 mL test solution. Tests were replicated three times for each concentration. Food was not provided for the larvae during the test period. Dead larvae were counted after a 24 h exposure.

### 2.5. Repellent Test

The repellent test was based on the WHO protocol (2009) [18] with a few modifications. The test was performed in a metal cage (30 cm × 20 cm × 20 cm) covered with a net. Fifty females (5–7 days old) were raised together with males to ensure copulation. They had no previous blood meal and had been fed on a sucrose solution of 10%. These female mosquitoes starved for 12 h before the tests. Two mosquito cages were randomly assigned to each participant. One cage was used for testing the essential oil solution and the other for the positive control (20% DEET standard solution in ethanol). The essential oil was tested at 25%, 50%, 100% concentration. In addition, a test solution containing the essential oil (25%) and vanillin (5%) was performed. Both forearms were covered with a rubber sleeve except a rectangle area (3 cm × 10 cm), thus the skin within these rectangle areas were directly exposed to the mosquitoes. Before each test, the forearms were treated with negative control (only ethanol) in order to ensure host-seeking behavior. On the forearms, each rectangle area was treated with 0.1 mL of repellent (essential oil solution or DEET 20% solution). After that, the protection time was considered started and each treated forearm was then inserted into a different cage for 3 min. If there was no mosquito bite, the test was repeated after a 30-minute interval. Once a mosquito sucked blood, the repellent test was considered finished. New mosquitoes were used for each test. Four volunteers who worked at the Faculty of Pharmacy and had no history of allergic reactions to mosquito bites were recruited. Following WHO’s guideline [18], we selected equal numbers of female and male volunteers. To avoid the problem of the limited number of volunteers, each participant was tested twice on different days to confirm the results. 

### 2.6. Data Analysis

Data analysis was performed by using the SPSS software program (IBM, version 22.0, Armonk, NY, USA). The larvicidal assay data were analyzed by probit analysis [19]. The probit-log(concentration) regression model was used to calculate LC_50_ values and 95% confidence limits. The differences in mean protection times among concentrations were analyzed by one-way ANOVA and Tukey’s post-hoc test, *p* < 0.05 was considered statistically significant. 

## 3. Results and Discussion

### 3.1. Yields and Chemical Constituents of the Essential Oil

The essential oil obtained from the hydro-distillation of *Mentha arvensis* L. leaves was achieved after one-hour extraction. The yield of the essential oil was 0.67% (v/w), calculated based on the fresh weight of the leaves. Table 1 shows the chemical composition of *Mentha arvensis* L. essential oil. The oxygenated monoterpenes (99.51%) was the major terpenoid group, including the main components such as menthol (66.04%), menthyl acetate (22.19%), menthone (2.51%), etc. Hydrocarbon monoterpenes (2.86%) and sesquiterpenes (0.90%) were the other terpenoid groups in lower proportion. There were thirteen main components in the essential oil in which menthol (66.04%) was the most abundant. This result was in good agreement with previous studies where menthol was also the most abundant component in the essential oil extracted from *Mentha arvensis* L. grown in Cuba (51.68%) [20], India (71.1%) [21], and Pakistan (~80%) [22], etc. The essential oil of *Mentha arvensis* L., also known as corn mint oil, is the main source of natural menthol for food and pharmaceutical industries.

### 3.2. Larvicidal Activity

Table 2 shows the toxicity of the essential oil against *Aedes aegypti* larvae. No mortality was seen in the control, while all larvae died at a concentration of 120 ppm. The LC_50_ and LC_90_ were 78.1 and 125.7 ppm, respectively. 

In a recent review, Pavela [23] has found that most essential oils with the value of LC_50_ less than 100 ppm against mosquito larvae were from five botanical families: Myrtaceae, Apiaceae, Rutaceae, Cupressaceae, and Lamiaceae. The genus *Mentha*, which belongs to Lamiaceae family, is cultivated over the world, and their oils have shown larvicidal effects on *Aedes aegypti* larvae [24,25,26]. However, the studies on the larvicidal effect of *Mentha* genus against *Aedes aegypti* larvae was limited to *Mentha piperita* [24], *Mentha x villosa* [25], and *Mentha spicata* [26]. In these previous studies, the LC_50_ value of *Mentha piperita* oil was 98.7 ppm [24], while the *Mentha spicata* oil and *Mentha x villosa* oil showed lower LC_50_ values of 56.1 ppm, 45.0 ppm, respectively. In this study, the LC_50_ value of *Mentha arvensis* essential oil was 78.1 ppm. In comparison with the essential oils, the synthetic chemical Temephos had a much lower LC_50_ value of 0.043 ppm [25]. Although *Mentha arvensis* L. essential oil shows the larvicidal effect against *Aedes aegypti*, further investigations on field application are neccessary.

Regarding the mode of action, essential oils can produce neurotoxic effects on insects through several targets such as inhibiting acetylcholinesterase enzyme in the cholinergic system [27] or acting on Octopamine receptor [28,29] and GABA receptor [30]. Furthermore, the synergistic effects of essential oil components probably increase the toxicity of the essential oil. Hummelbrunner et al. [31] found that thymol acted synergistically with trans-anethole to enhance the acute toxicity to tobacco cutworms. Osanlo et al. [32] found that the clove oil was more toxic than its major component eugenol. They suggested that the minor compound might act as a synergist that enhance the toxicity of the major compound. Santos et al. [33] reported that menthol had an LC_50_ value of 404 ppm against *Aedes aegypti* larvae. This value was much higher than LC_50_ value of 78.1 ppm of the *Mentha arvensis* essential oil, which contained menthol as the major component in this study.

### 3.3. Repellent Activity

**Figure 1:**
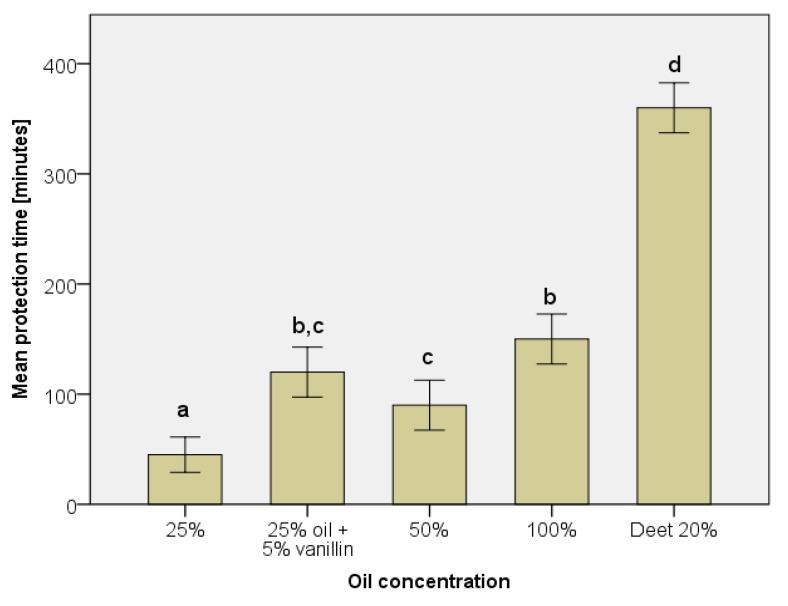
Protection times of *Mentha arvensis* L. essential oil against *Aedes aegypti* mosquitoes. Data are shown as mean ± SD (*n* = 4). Letters are used to show statistical significance (*p* < 0.05). Bars are significantly different if they do not share letters.

Figure 1 shows the repellency of the essential oil at different concentrations in ethanol compared with DEET 20%. The repellency of the essential oil increased with the concentration. The mean complete protection times were 45, 90, and 165 min at the concentration of 25%, 50%, and 100%, respectively. When adding 5 % vanillin to the essential oil (25 %), the complete protection time of the oil increased up to 120 min. However, the essential oil had a short protection time compared with Deet 20% (360 min). Campbell et al. [34] found that forty-two compounds in eleven essential oils elicited antennal responses from *Aedes aegypti*. These compounds were also found in the *Mentha arvensis* essential oil such as menthol, menthyl acetate, menthone, caryophyllene, and β-pinene.

The use of essential oils such as citronella oil, lemongrass oil, and eucalyptus oil is widely accepted as mosquito repellents [11]. Mosquitoes locate the human host by integrating olfactory, thermal, and visual cues. Among these, odors play an important role in human host detection [35]. Mosquitoes detect human odors such as CO_2_, acid lactic, and 1-octen-3-ol when that volatile odors bind to their odor receptors. The mode of action of mosquito repellents remains a controversial topic in which they may activate receptors associated with repellency or inhibit receptors associated with attraction [36]. 

Essential oils might produce effects through interaction with odor receptors, therefore decreases contacts between mosquitoes and their hosts. However, due to their high volatility, most essential oils have short repellent action, as compared with synthetic DEET [37]. To solve this problem, Khan et al. [38] used vanillin as a fixative to increase their protection time. Table 3 shows a review of the previous studies that reported the protection time of essential oils with and without vanillin. Tawasin et al. [39] found that an addition of 5% vanillin to citronella oil could significantly increase the protection time against *Aedes aegypti* up to 6.5 h. Songkro et al. [40] compared the evaporation rate of the citronella oil with and without vanillin. They found that citronella oil with vanillin had a lower rate of evaporation. Adding vanillin to *Zanthoxylum piperitum* [41], *Curcuma longa*, *Eucalyptus globulus*, *Citrus aurantium* [42], and lemongrass oil [43], etc was also reported to increase their protection times. However, if only 5% vanillin solution was tested, the protection time was 15 min [43]. Therefore, vanillin has acted as a synergist to those essential oils to enhance their repellency. In the present study, we found that the addition of 5% vanillin to the *Mentha arvensis* essential oil (25%) increased the protection time of the oil nearly 3 times, from 45 min up to 120 min. Recently, nanotechnology has been applied to slow the release rate of essential oil and thus prolong the protection time. Sakulku et al. [44] developed citronella oil nanoemulsion and found that the release rate of the essential oil in the nanoemulsion decreased and thus its protection time increased. Nuchuchua et al. [45] developed a nanoemulsion containing citronella oil, hairy basil oil, and vetiver oil. They found that this nanoemulsion increased the protection time up to 4.7 h. Although EO of *Mentha arvensis* L. shows promising repellency, incorporating the EO and vanillin into a nanoemulsion might extend their mosquito protection time. 

## 4. Conclusions

Our findings suggest that *Mentha arvensis* L. essential oil exhibits toxicity to the larvae of *Aedes aegypti*, which may be considered as a potential larvicide for controlling mosquito population. In addition, the EO shows excellent repellency against *Aedes aegypti* mosquitoes when adding 5% vanillin. Further studies on field applications as well as new repellent formulations based on the essential oil are necessary.

## Figures and Tables

**Table 1:** Chemical constituents (%) of *Mentha arvensis* L. essential oil.

No	RT (min)	Formula	Mass	Compound	Percentage (%)	Terpenoid Group
1	7.4	C_10_H_16_	136	α-Pinene	0.34	hydrocarbon monoterpenes
2	9.14	C_10_H_16_	136	Sabinene	0.09	hydrocarbon monoterpenes
3	9.23	C_10_H_16_	136	β-Pinene	0.39	hydrocarbon monoterpenes
4	11.93	C_10_H_16_	136	Limonene	2.04	hydrocarbon monoterpenes
5	19.22	C_10_H_18_O	154	Menthone	2.51	oxygenated monoterpenes
6	19.73	C_10_H_18_O	154	Isomenthone	1.45	oxygenated monoterpenes
7	19.83	C_10_H_20_O	156	neo-Menthol	1.89	oxygenated monoterpenes
8	20.48	C_10_H_20_O	156	Menthol	66.04	oxygenated monoterpenes
9	20.75	C_10_H_20_O	156	Isomenthol	0.2	oxygenated monoterpenes
10	23.69	C_10_H_16_O	152	Piperitone	1.47	oxygenated monoterpenes
11	25.26	C_10_H_22_O_2_	204	Menthyl acetate	22.19	oxygenated monoterpenes
12	29.15	C_15_H_24_	204	Caryophyllene	0.59	hydrocarbon sesquiterpenes
13	33.32	C_15_H_24_O	220	Caryophyllene oxide	0.31	oxygenated sesquiterpenes

RT: Retention times (minutes).

**Table 2:** Larvicidal activity of *Mentha arvensis* L. essential oil against *Aedes aegypti* larvae after 24 h exposure.

Concentration (ppm)	Mortality (%) ± SD	Slope (± SE)	LC_50_ ppm (CL 95%)	LC_90_ ppm (CL 95%)	χ2 (df)
0 (control)	0				
45	10.7 ± 4.6				
60	18.7 ± 4.6	6.201	78.1	125.7	24.9
75	45.3 ± 20.5	(± 0.630)	(72.0–85.4)	(109.4–160.5)	(13)
90	58.7 ± 16.7				
105	85.3 ± 8.3				
120	100				

SD = Standard deviation, LC_50_ = lethal concentration that kills 50% of larvae, LC_90_ = lethal concentration that kills 90% of larvae, CL = confidence limits at 95%, SE = standard errors, χ2 = chi-square value, and df = degrees of freedom.

**Table 3:** A review of protection times of essential oils with or without vanillin, against *Aedes aegypti* mosquitoes.

Essential Oils	Volume/Oil Concentration	Vanillin Concentration	Test Method	Area/Part Treated	Protection Time (Without—With Vanillin)	Ref.
*Mentha arvensis*	0.1 mL of 25% in ethanol	5%	Arm-in- Cage	30 cm^2^/forearm	45–120 (min)	This study
*Tumeric*	0.1 mL of 25% in ethanol	5%	Arm-in- Cage	30 cm^2^/forearm	1.0–4.0 (h)	[39]
*Kaffir lime*	0.1 mL of 25% in ethanol	5%	Arm-in- Cage	30 cm^2^/forearm	1.0–3.5 (h)	[39]
*Citronella*	0.1 mL of 25% in ethanol	5%	Arm-in- Cage	30 cm^2^/forearm	3.0–6.5 (h)	[39]
*Hairy basil*	0.1 mL of 25% in ethanol	5%	Arm-in- Cage	30 cm^2^/forearm	3.0–6.5 (h)	[39]
*Zanthoxylum piperitum*	0.1 mL of pure oil	10%	Arm-in- Cage	30 cm^2^/forearm	1.0–2.5 (h)	[41]
*Curcuma longa*	0.1 mL of 25% in coconut oil	5%	Arm-in- Cage	30 cm^2^/forearm	1.5–2.5 (h)	[42]
*Eucalyptus globulus*	0.1 mL of 25% in coconut oil	5%	Arm-in- Cage	30 cm^2^/forearm	66–144 (min)	[42]
*Citrus aurantium*	0.1 mL of 25% in coconut oil	5%	Arm-in- Cage	30 cm^2^/forearm	66–120 (min)	[42]
*Cassia oil*	0.1 mL of 5% in ethanol	5%	Arm-in- Cage	24 cm^2^/forearm	75–135 (min)	[43]
*Rosemary*	0.1 mL of 5% in ethanol	5%	Arm-in- Cage	24 cm^2^/forearm	0–52 (min)	[43]
*Lemon eucalyptus*	0.1 mL of 5% in ethanol	5%	Arm-in- Cage	24 cm^2^/forearm	22.5–60.0 (min)	[43]
*Xanthoxylum*	0.1 mL of 5% in ethanol	5%	Arm-in- Cage	24 cm^2^/forearm	30–60 (min)	[43]
*Lemongrass*	0.1 mL of 5% in ethanol	5%	Arm-in- Cage	24 cm^2^/forearm	30–105 (min)	[43]
